# National Cervical Cancer Screening in Thailand

**DOI:** 10.31557/APJCP.2021.22.1.25

**Published:** 2021-01

**Authors:** Pattama Ploysawang, Jinda Rojanamatin, Somjit Prapakorn, Paphawin Jamsri, Parinda Pangmuang, Kanda Seeda, Suleeporn Sangrajrang

**Affiliations:** *National Cancer Institute, Department of Medical Services, Ministry of Public Health Rama VI road, Ratchathewi Bangkok 10400, Thailand. *

**Keywords:** Cervical cancer, population-based organized screening, Thailand

## Abstract

**Background:**

Cervical cancer is an important public health problem in Thailand. It was the most common cancer in Thai women with the incidence rate of 23.4 per 100,000 women in 1990.

**Objective:**

The aim of this study was to share the experiences and summary the outcome of cervical cancer screening program in Thailand. Methods: The Ministry of Public Health in cooperation with the National Health Security Office, launched the National Cervical Cancer Screening Program, covering 76 provinces nationwide under Universal Coverage Scheme in 2005. The screening method are Pap smear and Visual Inspection with Acetic acid (VIA) for women aged 30-60 and 35-45 respectively with a 5-year screening interval. Detecting cervical pre-cancerous lesions will follow by day care treatment such as cryotherapy, Loop Electrosurgical Excision Procedure, etc.

**Results:**

The first phase (2005-2009), was carried out on 3,124,855 women, the coverage reached 77.5%. For the second phase (2010-2014), 7,637,226 women were screened, reaching 53.9% coverage of target women. However, we have few data of follow up examination after abnormal screening. Therefore, we conducted new system to get more follow up data in 2019. Under the coordination of many related partners, 10,762,081 women have been screened during 2005-2014. The incidence rate declined to 11.7 per 100,000 women which is ranked as the third most common in women in 2014.

**Conclusion:**

This article briefly reviews the challenge of implementing an efficient cervical cancer screening in Thailand. In 2020, HPV testing has been introduced as a primary screening test for all Thai women attending public health sector instead of conventional Pap test.

## Introduction

Cervical cancer is the fourth most common cancer among women worldwide with an estimated 570,000 new cases in 2018 representing 6.6% of all female cancers (World Health Organization, 2020a). In Thailand, cervical cancer is a major public health problem. According to data from the National Cancer Registry, there were an age-standardized incidence rate (ASR) of 23.4 per 100,000 in 1990 (Vatanasapt et al., 1993), affecting quality of life in Thai people and leading to a great loss of human and economic resources. Cervical cancer screening is effective at reducing morbidity and mortality (Hakama et al., 1985 ; Miller et al., 1990), it can detect the disease at an early stage or pre-cancer stage and greatly increases the chances for successful treatment outcomes and improved survival of patients. Also, early stage cancer treatment is significantly less expensive than treatment for advanced stage.

In view the national perspective, there are a range of different issues and challenges to implement an effective screening program as a public health policy, for example, financial and organizational frameworks, participation of target population, healthcare practitioners and infrastructure resources (World Health Organization, 2020b). Especially in Low Medium Income countries (LMICs), the challenges exist in poverty, not only in terms of limited access to health information or lack of knowledge of cervical cancer (Islam et al., 2017) but also the Socio-religious and cultural barriers may play an important role (Devarapalli et al., 2018). The World Health Organization (WHO) recommends that the screening methods should be made at the discretion of the available resources of the country or hospital, to be effective and maximize value (World Health Organization, 2020b). In Thailand, the national cervical cancer screening was established in 2005 by the Ministry of Public Health (MOPH) using Pap smear and Visual Inspection with Acetic acid (VIA) methods. The program provides the sustainable national comprehensive cervical cancer control program that allow equitable access to cervical cancer services for all Thai women. In this article, we briefly review existing experiences, achievements, constraints, and lessons learned from implementing the cervical cancer screening program in Thailand.

## Materials and Methods

*The pilot implementation model*

In 1999, National Cancer Institute (NCI), Department of Medical Service, Ministry of Public Health, initiated a pilot cervical cancer screening program at Nakhon Phanom Province, Thailand. This project is designed to determine a potential model for nationwide implementation (Deerasamee et al., 2007) such as available financial resources, structure and strategy of service providers, possible for scaling-up across country including early treatment services. 

The target population of the pilot project was women aged 35-54 years from 12 districts covered by 148 Primary Health Centers in Nakhon Phanom province. A total 32,632 women were screened by Pap smear for cytological examination. The detection of CIS and CIN III was obvious with a risk ratio of 6.9 (95% CI: 3.5-15.0) times higher among screened than non-screening target women. The screening program showed a considerable increase in early carcinoma in situ cases and should reduce incidence of and mortality from cervical cancer (Deerasamee et al., 2007). The results of this project has been evaluated for economic value by The Health Technology Assessment Policy (HITAP) (Praditsitthikorn, 2013). It was found that the project is cost effective and worth investing in Thai women to scaling up in the country.

*Preparation for components of screening process*

*Project plan meeting*

After the political decision has been taken to start the process of establishing a national program, the first step was setting up the meeting of representatives from various stakeholder groups to ensure that everyone was moving in the same direction. All stakeholders take this opportunity to discuss and exchange opinions, co-create an implementation plan and strategic road map. Moreover, they also have clearly defined the roles and responsibilities of local, regional and national program teams. At the same time, the infrastructure has been developed for comprehensive information system and collaboration between screening, diagnosis and treatment in each health area. 

*Training*

The expansion of national program across the country brings additional workload. The sufficient numbers of professional health worker is an essential for the delivery of quality health services to the population. NCI together with medical societies and provincial hospitals conduct the training of health professionals responsible for community education (nurses, doctors, and community health workers) in all regions of Thailand. 

Moreover, NCI and its affiliated organizations also provide of various educational materials to regional areas. The staffs from the Health Promotion Hospital (HPH) and the Village Health Volunteers play a major role in distributing cervical cancer knowledge and convincing targeted population about how important of screening. More than 1,000,000 Village Health Volunteers who serve as the backbone of Thailand’s primary health care system were very active on deliver the public understanding of the benefits and risks of cervical cancer screening. The ratio of one volunteer was responsible for ten houses.

*Software development*

NCI developed the software called “Cervical Cancer Screening program; CXS2010” to monitor the progress and evaluate the outcomes of the organized cervical cancer screening program. This program can be recorded screening, diagnosing and treatment data and the information can be link at the national level. It helps facilitates a routine procedure, improves the quality of the services provided, and manages several types of information.

*Implementation of the national cervical cancer screening program*

In 2005, the NCI, in cooperation with the National Health Security Office, launched the National Cervical Cancer Screening Program, covering 76 provinces nationwide under Universal Coverage Scheme. The screening method are Pap smear and Visual Inspection with Acetic acid (VIA) for women aged 30-60 and 35-45 respectively with a 5-year screening interval. The target women are able to access free cervical cancer screening at any HPH, which has nearly 10,000 sites nationwide. 

The screening was performed at the HPH, or mobile units, taking into account the monitoring of both the collection of samples and the laboratory analysis. All sample analyses were performed in the laboratory, consideration of variables such as the positivity rate, percentage of precursor lesions, the atypical squamous cell (ASC), squamous intraepithelial lesion (SIL), and percentage of diagnosis of atypical cells of undetermined significance (ASCUS). Then health care providers reported the results of cytological examination to screened women. Patients with abnormal tests are recommended to schedule a colposcopy appointment. The screening program also arranged the referral system for treatment of women who were diagnosed with precancers or cancer.

Clinical information such as the results of previously performed cervical cytology screening, adequacy of collected samples, follow-up, and colposcopy test were recorded in CXS2010 software. The system can identify each individual screening date and was able to automatically generate the due date for the next screening that bases on the previous cytological report and the time interval. The exams are routinely done at intervals of 5years. The recorded data are evaluated monthly, taking into consideration the quality of the collection in each health care unit or municipality. 

*Data analysis*

The calculation of incidence rates was based on the number of new cases registered at each registry. The age-standardized incidence rate is calculated first by estimating the age-specific incidence rates (Number of new cancer occurring in the age group*100,000 / Population of the age group) and then applying these rates to the standard population. The world standard population is used and expressed per 100,000 populations. The national estimate was pooled data from each region by adding together the cases and the person-years at risk. Then the age (five-year age groups) and sex specific rate for each region were calculated.

## Results

*Outcomes of Phase 1 (2005 - 2009)*

The first five years, there was focused on the comprehensive management plan of screening program including building up health professionals, increase public understanding and offer CXS2010 software consulting services. This phase was examined 3,124,855 women out of totally 4,030,833 target population participated in the screening with the participation rate of 77.52%. Women with histopathological diagnosis of cancer, high-grade squamous intraepithelial lesions (HSIL) and low-grade squamous intraepithelial lesions (LSIL) were 0.1%, 0.5% and 0.9% respectively. The number and percentage distribution of cytological diagnosis result were shown in Table 1.

*Outcomes of Phase 2 (2010 - 2014)*

The screening program in phase 2 has set a target of 9,577,584 women. There were 7,637,226 screened women, accounting for 79.74% of the target. Out of these, 2,472,475 women were scheduled to repeat the exam within 5 years, therefore the percentage of women who received the screening 1 in 5 years was only 53.92%. The results showed that abnormal cases were found in 1.3%, consisting of suspected cancer, HSIL and LSIL were found in 0.05 %, 0.40 % and 0.88 % respectively (Table 2). Thai Society of Cytology supported the quality assurance of the project by reviewing and reporting on randomly selected 2.3-8% in each year of normal slides from all laboratories across the country. It was found that the false negative rate (FNR) is similar to the result, 17-61%, of other countries (Anderson et al., 1992; Koss et al., 1997; Ejersbo et al., 2003; Castillo et al., 2016), as shown in Table 3. The percentage of abnormal screening reports in terms of cancer, HSIL and LSIL in each year were similar, reflecting the consistent quality of screening.

In this phase, we also focused on follow-up of abnormal screening results, women receiving an abnormal result are given follow-up diagnostic recommendations colposcopic examination. However, from 101,555 abnormal women reporting (Table 2) revealed that only 9,582 women (9.44%) obtained to follow-up care. Of those who received follow-up care, 5092 (5.01%) received diagnostic colposcopy. We investigate why the follow up was so low despite the follow up procedure or treatment cover by Universal Coverage with free of charge services. Then we conducted a field study in 4 provinces in different areas. The results show that 66.15% of abnormal results received the repeat Pap smear in cases the cytology results was LSIL and the abnormal results over HSIL were referred to have further investigate with colposcopy in the hospitals (Table 4). We organize the meeting and informed health personals who involved in the project about how important to record the follow up results. 

**Figure 1:**
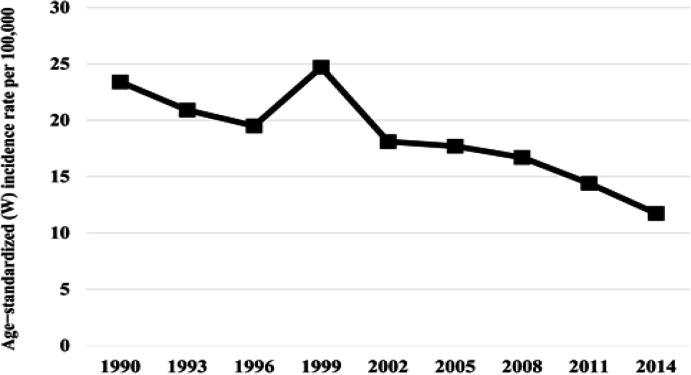
Trends in the Age-Standardized Incidence Rates for Cervical Cancer in Thailand, 1990-2014. 1990 (Vatanasapt et al., 1993), 1993 (Deerasamee et al., 1999), 1996 (Sriplung et al., 2003), 1999-2008 (Khuhaprema et al., 2007; 2010; 2012; 2013), 2011 and 2014 (Imsamran et al., 2018)

**Figure 2:**
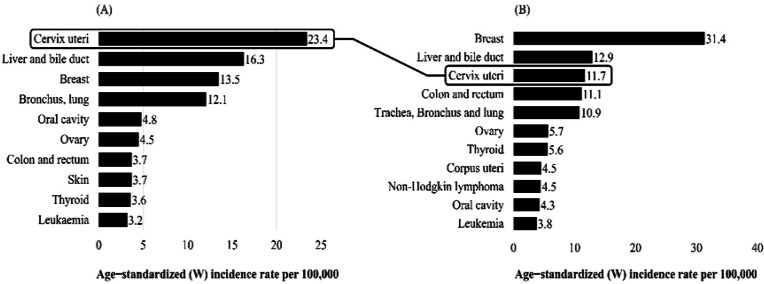
Age-Standardized Incidence Rates of the Ten most Frequent Cancers in Thai Women, (A) 1990 (Vatanasapt et al., 1993) and (B) 2014 (Imsamran et al., 2018)

From 2005 until 2014, there were 10,762,081 target women who participated in the screening program at least once. The overall screening coverage was more than 70%, which was variation by geographic areas and population group with different socio-economic background. Trend analysis of the Age-Standardized incidence rate showed declines over the years (Figure 1), from being the most common cancer in Thai women in 1990, cervical cancer is now ranked as the third most common in women with the incidence rate 11.7 per 100,000 women (Imsamran et al., 2018) (Figure 2).

**Table 1:** Cervical Cytology Results During 2005-2009

	Fiscal year of screening					
	2005	2006	2007	2008	2009	Total
Total of screened	405,756	586,981	855,090	642,155	634,873	3,124,855
Number of abnormal (%)	3,169 (0.78)	9,800 (1.67)	14,795 (1.73)	9,205 (1.43)	8,910 (1.4)	45,879 (91.47)
-LSIL (%)	1,814 (0.4)	5,645 (1.0)	8,834 (1.0)	5,524 (0.9)	5,436 (0.9)	27,253 (0.9)
-HSIL (%)	1,141 (0.3)	3,541 (0.6)	5,116 (0.6)	2,994 (0.5)	2,914 (0.5)	15,706 (0.5)
-Cancer (%)	214 (0.1)	614 (0.1)	845 (0.1)	687 (0.1)	560 (0.1)	2,920 (0.1)

**Table 2:** Cervical Cytology Results During 2010-2014

	2010	2011	2012	2013	2014	Total
Total of screened	2,168,248	1,713,490	1,500,413	1,247,051	1,008,024	7,637,226
Number of abnormal (%)	24,396 (1.13)	22,681 (1.32)	21,460 (1.43)	17,545 (1.41)	15,473 (1.53)	101,555 (1.33)
Low grade (%)	15,012 (0.69)	14,825 (0.87)	14,630 (0.98)	12,015 (0.96)	10,643 (1.06)	67,125 (0.88)
High Grade (%)	8,326 (0.38)	7,035 (0.41)	6,096 (0.41)	4,958 (0.4)	4,346 (0.43)	30,761 (0.4)
Cancer (%)	1,058 (0.05)	821 (0.05)	734 (0.05)	572 (0.05)	484 (0.05)	3,669 (0.05)

**Table 3:** Number of Rescreened Slides and False Negative Rate among Negative Results, 2010-2014

	2010	2011	2012	2013	2014
Number of slides	2,168,248	1,713,490	1,500,413	1,247,051	1,008,024
Abnormal results	24,396	22,681	21,460	17,545	15,473
Normal results	2,143,852	1,690,809	1,478,953	1,229,506	992,551
Number of rescreened slides (%)	50,005 (2.3)	50,070 (2.9)	100,000 (6.7)	100,000 (8.0)	30,000 (3.0)
Number of qualified slides	49,414	49,256	97,933	98,006	29,458
Number of unsatisfied slides	591	814	2,067	1,994	542
False negative (%)	434(0.9)	412(0.8)	468(0.5)	579(0.6)	187(0.6)
Percent of false negative rate	43.3	38	24.4	28.9	28.6

**Table 4:** Follow-up of Patients with Abnormal Pap Test Results in 4 Provinces

Province	Pap smear result (cases)		Follow up	Further investigation	
			(cases, %)	Repeat Pap smear	Colposcope
Ubon Ratchathani	Low Grade	183	114 (62.30)	79	39
	High Grade	49	36 (73.47)	11	26
	Cancer	13	10 (76.92)	2	5
	Total	245	160 (65.31)	92	70
Ratchaburi	Low Grade	160	105 (65.63)	89	32
	High Grade	65	63 (96.92)	44	20
	Cancer	7	5 (71.43)	2	1
	Total	232	173 (74.57)	135	53
Suphanburi	Low Grade	115	59 (51.30)	14	0
	High Grade	58	33 (56.90)	6	0
	Cancer	7	5 (71.43)	1	0
	Total	180	97 (53.89)	21	0
‎Phitsanulok	Low Grade	24	17 (70.83)	6	11
	High Grade	37	25 (67.57)	15	17
	Cancer	11	9 (81.82)	3	6
	Total	72	51 (70.83)	24	34

## Discussion

Prior to 2005, cervical cancer screening was conducted as opportunistic screening in both public and private hospital. Only women who have high income or live in the city could access screening services. The national cervical cancer screening program provides equality of opportunities for all Thai women, including those who have low income and living in rural areas. 

However, there are various obstacles of program implementation. One of the most important is screening rate which caused by diversity of socio-religious and poverty within population. Many studies have revealed that the income, culture and lack of knowledge about screening which affect on participating of screening rate. (Underwood et al., 1999; Carroll et al., 2007; Al-Amoudi et al., 2013; Ferdous et al., 2018). Especially knowledge, that was the significant factor associated with a positive attitude towards cervical cancer screening (Tekle et al., 2020). Improving women ’s knowledge of cervical cancer can not only help them to understand and reduce their personal health risk of illness, but also reduce their anxiety during screening (World Health Organization, 2014). 

Other limitation of our program was the failure to follow up data on the abnormal results, less than 10 % of women with abnormal results receive follow-up care were recorded. Since 2015, we have changed the monitoring software program. The MOPH has been implemented the national health data in the form of database files, all services or procedures that have been done in HPH or in hospitals have to report to MOPH. We could link the screening, follow up procedure and treatment with the ID number of patients. 

In addition, new innovations such as mobile units, after-hour services, use of facial masks, privacy for pelvic examination and selection of female HPH staff to minimize embarrassment, are featured.

Over the past 15 years, one of the success and sustainable of the program is the awareness and understanding of cervical cancer screening among Thai women. During this national policy implementation period, we also encouraged women to underwent screening test through educational campaigns aimed at increasing awareness of cervical cancer screening. Currently, Thai women are more aware of screening’s benefits and more likely to participate in the national cervical cancer screening programs, consistent with the results of the survey that showed that 67.4% of women aged 30-60 years were screened for cervical cancer in the past 5 years in 2010 (Joseph et al., 2015).

In summary, we could overcome many difficulties even it took time to initiate the national cervical cancer screening program. This program is successful in cooperation with a multi-professional team. Sustainability is essential to achieve long-term health outcomes, so we intend to improve and maintain high-quality of cervical cancer screening service in Thailand. From 2020, HPV testing has been introduced as a primary screening test for all Thai women attending HPH instead of conventional Pap test.

